# First-Principles Thermodynamics of CsSnI_3_

**DOI:** 10.1021/acs.chemmater.2c03475

**Published:** 2023-02-06

**Authors:** Lorenzo Monacelli, Nicola Marzari

**Affiliations:** Theory and Simulation of Materials (THEOS), and National Centre for Computational Design and Discovery of Novel Materials (MARVEL), École Polytechnique Fédérale de Lausanne, 1015Lausanne, Switzerland

## Abstract

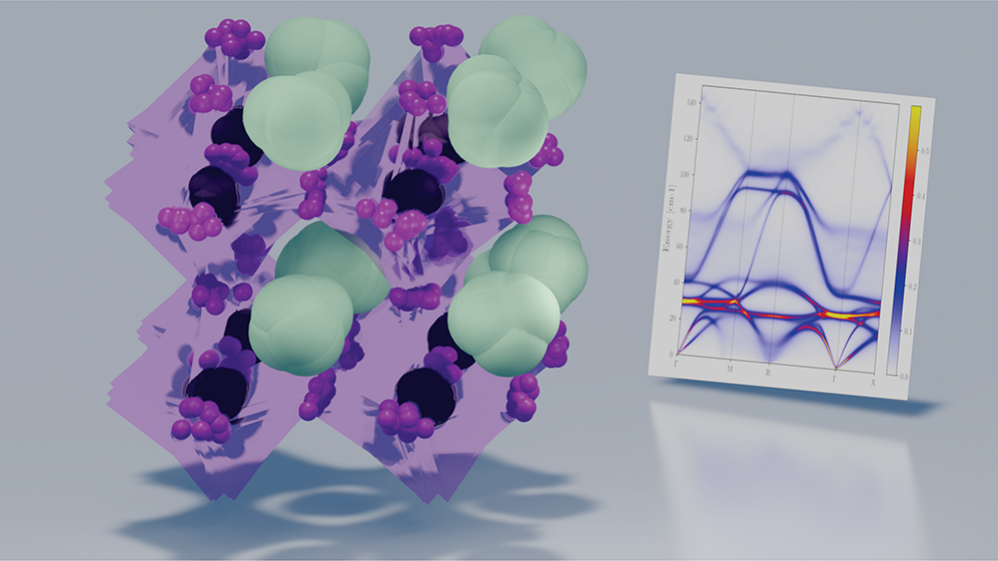

CsSnI_3_ is a promising ecofriendly solution for energy harvesting technologies. It exists at room temperature in either a black perovskite polymorph or a yellow 1D double-chain, which irreversibly deteriorates in the air. In this work, we unveil the relative thermodynamic stability between the two structures with a first-principles sampling of the CsSnI_3_ finite-temperature phase diagram, discovering how it is driven by anomalously large quantum and anharmonic ionic fluctuations. Thanks to a comprehensive treatment of anharmonicity, the simulations deliver a remarkable agreement with known experimental data for the transition temperatures of the orthorhombic, rhombohedral, and cubic perovskite structures and the thermal expansion coefficient. We disclose how the perovskite polymorphs are the ground state above 270 K and discover an abnormal decrease in heat capacity upon heating in the cubic black perovskite. Our results also significantly downplay the Cs^+^ rattling modes’ contribution to mechanical instability. The remarkable agreement with experiments validates our methodology, which can be systematically applied to all metal halides.

## Introduction

Perovskites have an ABX_3_ chemical formula, where the B-site cation is octahedrally coordinated in a BX_6_ configuration and the A cation sits within the cuboctahedral cavity formed by nearest-neighbor X atoms in an AX_12_ polyhedron. Metal-halide perovskites (MHPs), in particular, are typically composed of a divalent B-site metal (e.g., Pb^2+^, Sn^2+^, Ge^2+^, Cu^2+^, Eu^2+^, and Ni^2+^) and monovalent A-site cation. Inorganic MHPs usually employ Cs^+^ cations to improve stability. Among all, the inorganic cesium lead halide CsPbI_3_ has been considered the best candidate for solar cells applications due to its suitable band gap of 1.73 eV and excellent optoelectronic properties.^1^ First reported in 2014, CsPbI_3_ perovskite solar cells (PSCs) have achieved remarkable progress in stability and power conversion efficiency through additive and composition engineering, interfacial modifications, and optimization of the fabrication process.^2^ Nevertheless, the presence of toxic lead hampers its deployment into general markets. CsSnI_3_ has established itself as the most promising ecofriendly alternative.^3^

CsSnI_3_ is polymorphic with two different phases coexisting at room temperature. The first black phase (B) is a standard perovskite crystal, which goes through three different phase transitions upon heating: it transforms from B-γ (orthorhombic *Pnma* symmetry) to B-β (tetragonal *P*4/*mbm*) at 362 K and then to B-α (cubic *Pm*3̅*m*) at 440 K.^4^ The second phase appears when CsSnI_3_ is exposed to air at room temperature; under these conditions, B-γ transforms instead into a yellow phase (Y) with an orthorhombic *Pnma* space group, where the SnI_6_^–^ octahedra are connected into one-dimensional chains sharing one edge. In practice, CsSnI_3_ is synthesized at high temperature in the B-α phase.^4^ Then, when cooled to room temperature and exposed to air, it transforms into the yellow phase Y, suggesting that the perovskite structures (B-γ, B-β, and B-α) are metastable under ambient conditions.

All the B phases display excellent optoelectronic properties and are considered the most promising ecofriendly candidates for high-performance PSCs. On the contrary, the Y phase is easily oxidized and irreversibly transforms to Cs_2_SnI_6_ whose absorption coefficient is ten times lower than the black perovskite polymorphs.^5,6^ Notably, also other isostructural tin metal-halides have been found to decompose into the Y phase.^3^ Therefore, understanding the mechanical stability between the perovskite polymorphs and the Y phase is a fundamental step to improving the overall stability of the CsSnI_3_ and its practical deployment.

Due to the experimental difficulties in the production of single crystals in the B-γ phase, the structural characterization through X-ray spectroscopy has been achieved only recently,^4^ and our understanding of the B perovskite phase diagram is still in the early stages. For example, the isomorph compound CsPbI_3_ was shown to form small domains of the orthorhombic black phase B-γ within the cubic structure (B-α) even at high temperatures.^7,8^ So, it is not clear if the transition between ferroelectric B-γ, B-β, and paraelectric B-α in CsSnI_3_ is of the second-order displacive kind, where B-α is a high-symmetry homogeneous crystal; or an order–disorder phase transition, where the crystal displays a local electric dipole even in the paraelectric phase.^9^ Moreover, theoretical calculations failed so far in reproducing even qualitatively the phase diagram of CsSnI_3_, with the B-α phase predicted in many studies not to exist at any temperature.^10,11^ This contrasts with experiments, which observe the B-α phase above 440 K. Further studies tried to include anharmonicity in the calculations, which is essential to describe the ferroelectric transition,^12^ but the lack of algorithms to simulate lower-symmetry phases, introduced only recently,^13^ prevented the simulation of the complete phase diagram. Moreover, the relative stability between Y and perovskite phases is extremely challenging for first-principles molecular dynamics, as the transition involves a macroscopic structural rearrangement of atoms. Thus, the phase diagram of CsSnI_3_ remains largely unknown: Are the transitions between B-γ, B-β, and B-α displacive or order–disorder? Is B-γ or Y the lowest free energy phase at room temperature?

Here, we answer these questions by simulating the complete phase diagram of bulk CsSnI_3_ from first-principles using state-of-the-art sampling techniques and disclosing the origins of the formation of the Y phase. In the process, we elucidate the displacive character of the ferroelectric phase transitions in the black perovskite, highlight its anomalous heat capacity, and show the impact of the tin and cesium rattling motions on mechanical stability.

## Results

The importance of anharmonicity has been recently discovered in the isostructural compounds CsPbI_3_^14,15^ and CsPbBr_3_.^16,200^ As will be seen below, anharmonicity in thermal and quantum ionic fluctuations plays a crucial role in the thermodynamic properties and phase diagram of CsSnI_3_. We account for this by employing the stochastic self-consistent harmonic approximation (SSCHA),^17^ combined with density-functional theory (DFT) at the PBEsol level.^18^ The SSCHA, essential to this work, captures the strongly anharmonic fluctuations of the ions by optimizing a nuclear quantum distribution that minimizes the free energy.^19^ Within the SSCHA, one can optimize the average ionic positions (the centroids of the nuclear quantum distribution), the lattice vectors, and cell volume as a function of temperature. The method is stochastic and samples the energy landscape extracting configurations with randomly displaced ions and evaluating the respective energies and forces within DFT. The advantages of the SSCHA compared to other state-of-art approaches, like *ab initio* molecular dynamics, are the direct access to free energies, also exploiting symmetry constraints, and the inclusion of quantum nuclear zero-point motion. Moreover, unlike other approximate methods like the time-dependent energy landscape (TDEP^20^), it is nonempirical and has no internal free parameters that could affect the results (like the choice of the diagrams to include in the phonon self-energy or the order of the fit for the energy landscape).

**Figure 1:**
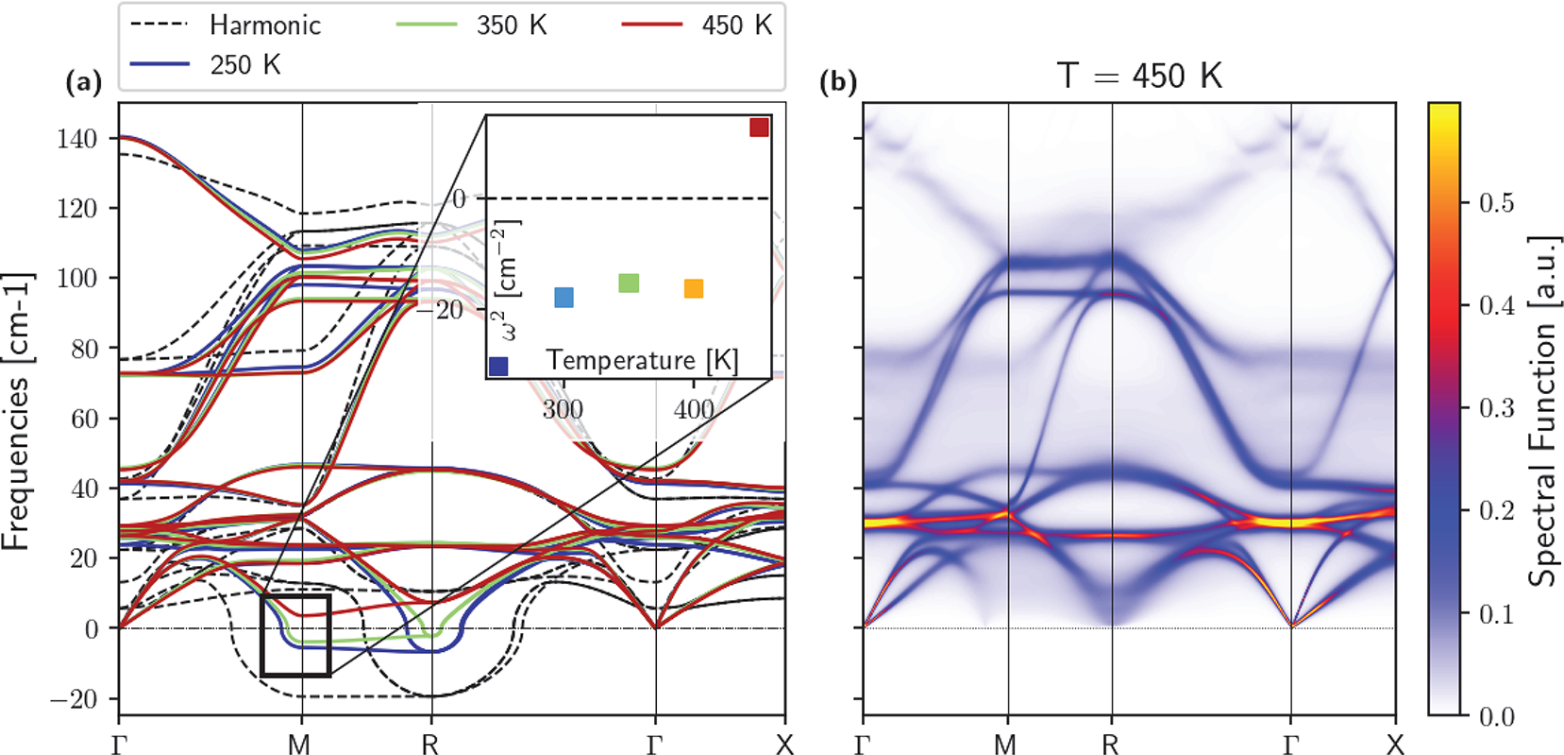
(a) Phonon dispersions of the B-α phase computed within the harmonic approximation (black dashed lines) contrasted with the full inclusion of anharmonicity within the SSCHA at different temperatures; negative values indicate unstable (imaginary) vibrations. In the inset, we report the second derivative of the vibrational free energy for the ionic displacement at M that transforms B-α into B-β. (b) Vibrational spectrum of B-α at 450 K. The finite width of the bands is given by physical lifetimes due to phonon–phonon scattering. Most phonon bands have extraordinary line widths leading to coherent thermal transport across different bands.^22^

The most simple structure for CsSnI_3_ is the standard cubic perovskite B-α (space group *Pm*3̅*m*), with five atoms in the primitive cell. Despite its geometrical simplicity, it is a saddle-point of the Born–Oppenheimer energy landscape, and the harmonic phonon dispersion presents imaginary frequencies (Figure 1a). Moreover, extrinsic thermal effects (e.g., volume expansion) further destabilize the structure, introducing an additional imaginary mode at Γ.^11^ To assess the stability of the B-α phase, we computed the Hessian of the free energy with respect to the centroids,^21^ which defines an effective anharmonic temperature-dependent static phonon dispersion. The critical temperature at which B-α becomes mechanically stable occurs when the phonon dispersion becomes positive in the whole Brillouin zone (Figure 1a); we report more details on the calculations in the Methods section. An ionic displacement with imaginary frequency in the M–R region of the Brillouin zone identifies the low-temperature B-α instability. The instability disappears at R between 350 and 400 K, then at M at 450 K as B-α becomes stable. This is in very good agreement with experiments that show how the B-β phase transforms into the B-α between 430 and 440 K.^4^ At variance with the unstable M–R phonon modes, other frequencies display only a slight variation with temperature, despite their substantial difference with respect to the harmonic spectrum. Such a strongly anharmonic character becomes even more apparent when examining the vibrational spectrum of the B-α phase. This is reported in Figure 1b and is evaluated as the trace of positions’ autocorrelation functions within the time-dependent self-consistent harmonic approximation (TD-SCHA)^17^ (more details in the Methods section); the very broad line width of the phonon bands points to their short lifetimes. The phonon–phonon scattering is so strong that the character of the dispersion disappears, and almost all phonons merge. This justifies a posteriori the necessity of dealing carefully with such strongly anharmonic crystals, and the overlap between different phonon bands points toward the importance of coherences and Wigner transport for thermal conductivity,^22^ neglected in the standard Boltzmann theory, and the necessity to account for the overdamped regime of the low-frequency modes.

**Figure 2:**
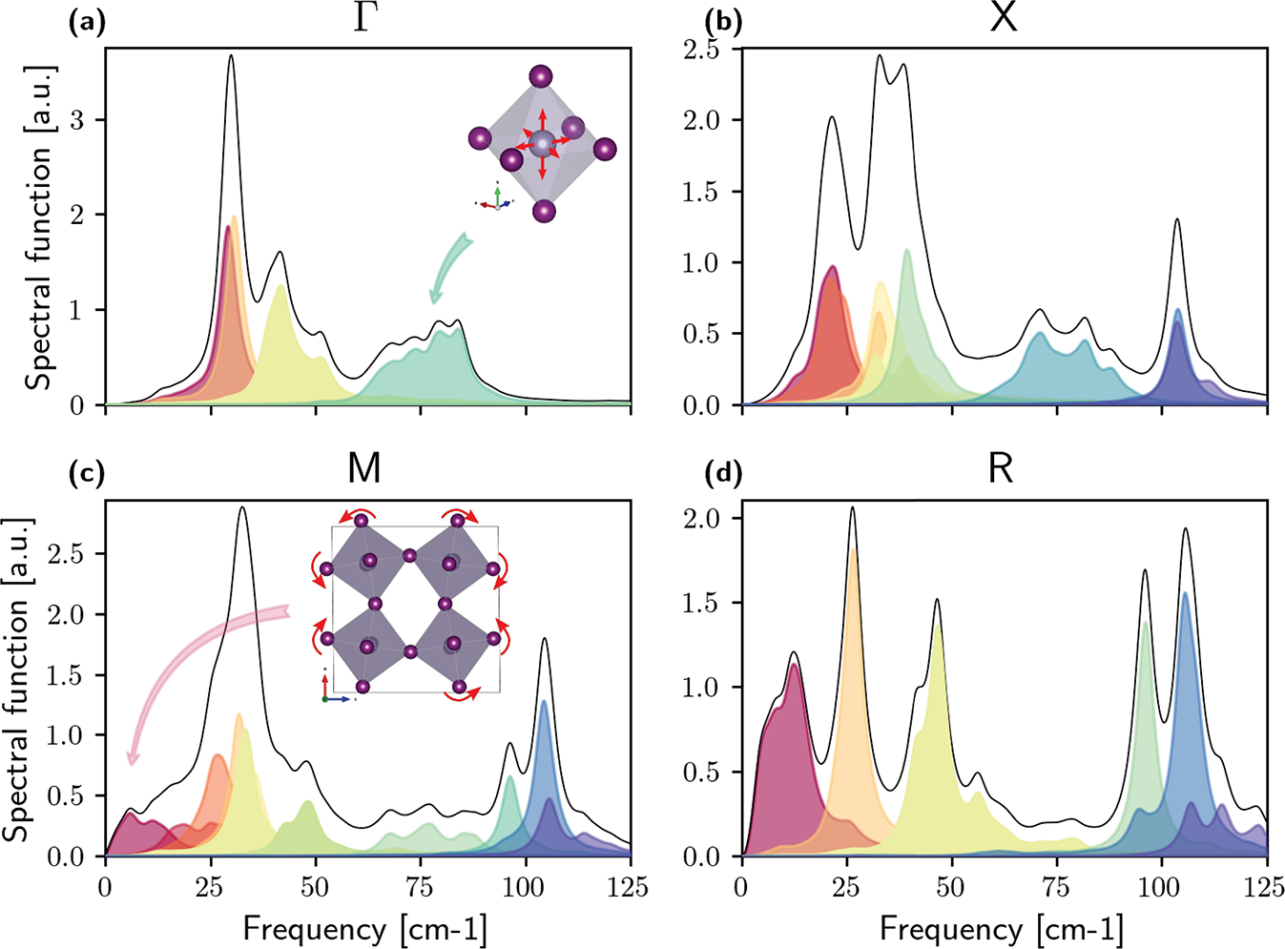
Spectral function of the B-α phase at 450 K and different high-symmetry points. The simulations are performed with a smearing of 5 cm^–1^; thus, the overall shapes of the peaks represent the intrinsic finite lifetimes due to phonon scattering. We report the single contribution of each phonon mode, highlighted with different colors. In panel a, we neglected the LO-TO splitting at Γ. Atomic vibrations for tin rattling are reported in the inset in panel a. The SnI_6_ octahedra tilting driving the phase transition from B-α to B-β is reported in panel c; a similar tilting is also present in R (d) at very low frequencies.

To further investigate the vibrational properties of the B-α phase, we dissected the spectral function separating the contribution of each mode in the Γ, M, R, and X high-symmetry points in Figure 2, including also the effects of four-phonon scattering self-consistently^17^ (see the Methods section).

Almost all phonon modes in the Brillouin zone display a peak shape departing from the standard Lorentzian. The mode which drives the phase transition between B-α and B-β is shown as a dark-red broad band at M and R (Figure 2c,d). This band represents the tilting of the SnI_6_^–^ octahedra along the different directions; it has a broad featureless spectrum spanning low frequencies up to 20 cm^–1^ and is strongly overdamped with a lifetime shorter than the oscillation period. A similarly broad spectrum has already been measured in the isostructural CsPbBr_3_.^23^ However, even higher energy modes show nontrivial peak shapes; e.g., the phonon branch around 75 cm^–1^ at Γ, X, and M has a large broadening of 30 cm^–1^. This is the mode for Sn rattling inside the SnI_6_ cages, underlining how temperature delocalizes the bonding of tin (Figure 2a–c). In contrast, the modes involving the motion of Cs^+^ ions have a Lorentzian shape (e.g., the one marked in red around 31 cm^–1^ at Γ, or the one in orange around 26 cm^–1^ at R; Figure 2a,d); this is a signature that atoms vibrate similarly to an *effective* harmonic oscillation.

**Figure 3:**
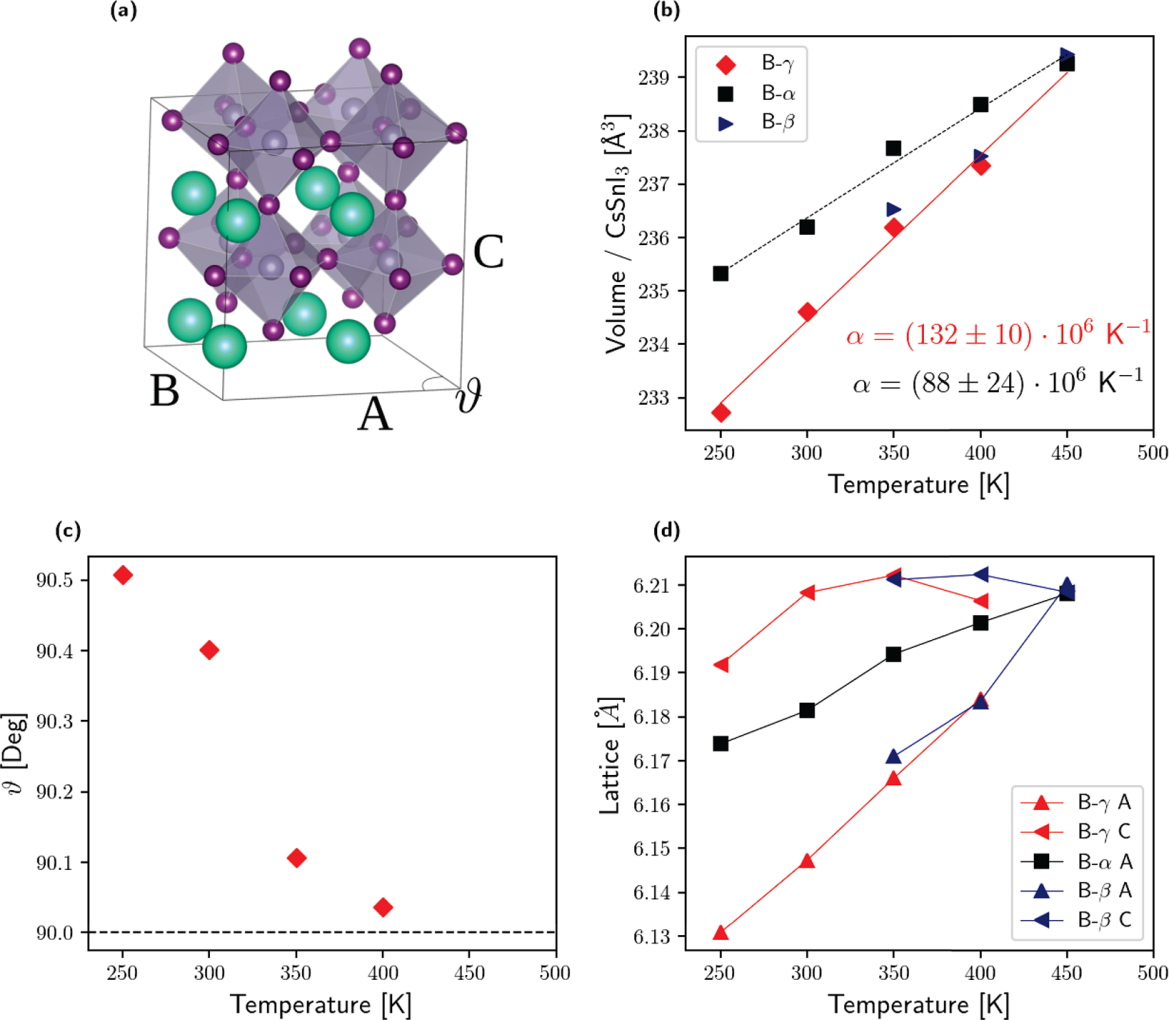
Structure of the black perovskite CsSnI_3_. (a) Conventional cell of 48 atoms commensurate with B-α, B-β, and B-γ (rendered with VESTA^25^). For cubic B-α, it is a 2 × 2 × 2 supercell. The primitive unit cell of B-β and B-γ is shown in red, identified by *a* and *b* and *C* segments. The ϑ angle is 90° when *a* = *b*: in the tetragonal B-β. When also *A* = *C*, the phase is cubic, as in B-α. (b) Volume expansion as a function of temperature; the thermal expansion coefficient α_v_ computed at 300 K is reported on the plot. (c) ϑ angle between *A* and *B*. (d) Size of the lattice parameters. Analyzing the lattice parameters ϑ, *A*, and *C*, we conclude that the structure transitions to the rhombohedral B-β between 350 and 400 K and to the cubic one B-α at 450 K. These transitions are confirmed by further symmetry analysis of the centroids.

These observations challenge the common assumption that Cs^+^ ions rattle inside the oversized octahedra of the perovskite structure^4,24^ and that this motion plays a crucial role in the stability of the perovskite structure, an assumption incorrectly corroborated by quasiharmonic simulations, which show that Cs^+^ vibrations become imaginary at the Γ point upon volume dilation.^11,24^ Indeed, Figure 1a shows a relevant frequency shift with respect to the harmonic value (from 5 to 28 cm^–1^ at 450 K); however, Cs^+^ motion is stable already at 250 K when intrinsic anharmonicity is accounted for and barely depends on temperature. Despite the agreement between our simulations showing the B-α phase becoming stable between 400 and 450 K and the experimental transition temperature, questions remain regarding the order of the phase transition (first or second) and its character (order–disorder or displacive). To answer these questions, we further investigated the ferroelectric transition in the B-γ and B-β phases: we prepared the starting trial nuclear density matrix in the orthorhombic B-γ phase and optimized the SSCHA distribution, including the cell shape and centroids within the symmetry constraints of the *Pnma* orthorhombic group from 250 to 450 K (each 50 K). The procedure is repeated with the symmetry constraints of B-β (*P*4/*mbm*). To detect the transition, we measure the distortion of the conventional cell with 48 atoms (commensurate with all the three phases, Figure 3a) in analogy with X-ray diffraction experiments. Namely, the orthorhombic to rhombohedral transition is identified by the ϑ angle between the lattice vectors of the almost cubic 48-atom supercell (Figure 3c) and the rhombohedral to cubic transition by the relative size of the lattice parameters *A* and *C* (Figure 3d). The deviation of ϑ from 90° (Figure 3c) shows how the B-γ phase transforms continuously into B-β between 350 and 400 K. The transition to the cubic B-α phase occurs at around 450 K, as shown by the value at which the cell becomes cubic (Figure 3d) and the temperature at which the volumes of the simulations constrained along the B-γ and B-β phases intersect the one of the B-α phase (Figure 3b). A symmetry analysis of the centroids confirms both transitions. The resulting phase diagram is in excellent agreement with experimental data (B-β at 362 K and B-α at 440 K^4^): the match between this simulation and the stability analysis of the phase B-α indicates that there is no metastability region for the B-α phase (no hysteresis between B-β and B-α), and it confirms a second-order phase transition supporting the displacive scenario, as the crystal has no local ferroelectricity above the critical temperature.

CsSnI_3_ displays a remarkably large thermal expansion coefficient  at room temperature of (94 ± 28) × 10^6^K^–1^ in the cubic B-α phase and (132 ± 10) × 10^6^ K^–1^ in the orthorhombic B-γ phase. This latter value is in excellent agreement with *in situ* diffraction experiments (126 × 10^6^ K^–1^ in the B-γ phase^4^), validating the accuracy of the PBEsol functional and our treatment of anharmonicity. These values are uncommon when compared with other materials: for example, the isostructural compound MgSiO_3_ has a α_v_ equal to 15 × 10^6^ K^–1^,^26^ while SrZrO_3_ and BaZrO_3_ have values of 29.8 × 10^6^ and 10.6 × 10^6^ K^–1^ respectively.^27^ The volumetric thermal expansion coefficient α_v_ of B-γ CsSnI_3_ is the largest known for a crystal, approaching those of amorphous systems and liquids.^4^

**Figure 4:**
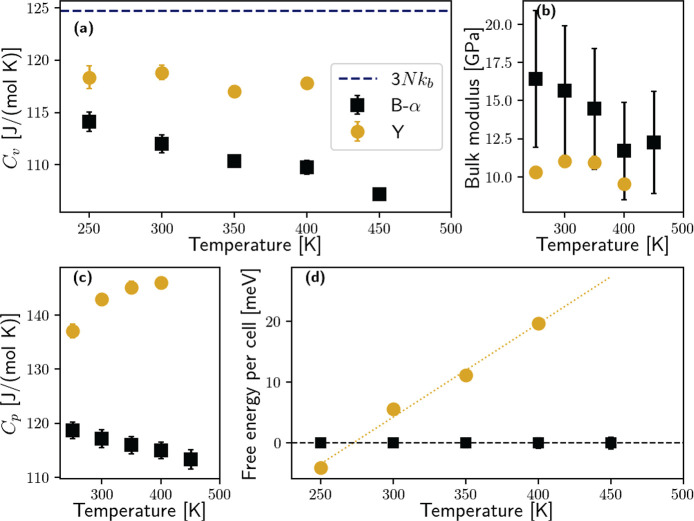
Thermodynamic properties of the B-α and Y phases of CsSnI_3_. (a) Constant-volume heat capacity. In a harmonic system, *C*_v_ should approach the classical value 3*Nk*_b_ (124.7 J mol^–1^ K^–1^) at high temperatures, reported here as a blue dashed line. However, B-α shows an anomalous heat capacity decrease upon heating, and also, Y does not converge to the expected classical value. (b) Bulk modulus. (c) Constant-pressure heat capacity. (d) Free energy difference between the B-α and Y phases. Above about 270 K, the B-α phase becomes favored. Here, B-α is also used as a prototype for B-β and B-γ, as their free energy differences are below 2 meV per formula unit in the whole temperature range studied.

Using the SSCHA we can compute the free energy at finite temperatures even in materials with strong intrinsic anharmonicity, as is the case for the B-α phase of CsSnI_3_. To shed light on the decomposition of this black perovskite into the orthorhombic yellow phase (Y) when the sample is exposed to air, we ran a new SSCHA calculation on the Y phase and compared its free energy and thermodynamic properties as a function of temperature with that of the B-α phase (Figure 4). Since the B-α, B-β, and B-γ phases transform through second-order phase transitions, their free energy differences are below 2 meV per formula unit in the temperature range studied (250–450 K). Therefore, we employed the B-α as a prototype for the all three black perovskites when assessing its relative stability with respect to the Y phase, as the SSCHA can reach a lower stochastic error and a better thermodynamic limit extrapolation exploiting the higher number of symmetries of the cubic phase.

We compare in Figure 4a–c the constant-volume heat capacity *C*_v_, the bulk modulus, and constant-pressure heat capacity *C*_p_ for the B-α and Y phases. Notably, as a function of temperature, the constant volume heat capacity (Figure 4a) of the B-α decreases anomalously. According to harmonic theory, above the Debye temperature *T*_*D*_ ≈ 230 K, *C*_v_ should reach 3*Nk*_b_ = 124.7 J mol^–1^ K^–1^ (blue dashed line in Figure 4a). The anomalous thermal dependence of the heat capacity in the B-α phase instead further underlines the anharmonicity of the crystal. In fact, according to the SSCHA (see the SI), the contribution of each phonon to the heat capacity is
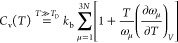
1where the first term is the standard Dulong–Petit model for solids (3*Nk*_b_), while the second one accounts for the intrinsic anharmonic shift of frequencies with temperature at constant volume; this is not captured by the quasiharmonic approximation. The B-α phase instability close to 450 K (Figure 1a) generates a softening of the full phonon branch between M and R, which, thanks to the 1/ω factor, enhances the effect of anharmonicity and explains the negative slope of the heat capacity before the phase transition.

The constant-volume heat capacity of the Y phase does not show the same anomaly: it is almost independent of temperature, and it deviates from the value predicted by the harmonic theory by 6%. The difference between the Y and B-α phases is further enhanced at constant pressure (Figure 4c), due to the higher thermal expansion coefficient of the Y phase [α_v_ = (117 ± 4) × 10^6^ K^–1^]. The bulk moduli of both phases are very similar, with a softer value for the Y phase at low temperature. The bulk modulus of the cubic perovskite structure shows a slight decrease with temperature, and its much smaller value when compared with other perovskite structures (e.g., *B* = 170 GPa in SrTiO_3_, about 15 times larger) is at the root of the remarkable softness of CsSnI_3_ and its sizable thermal expansion coefficient.

The free energy calculations unveil how the B-α phase is more stable than the Y above 270 K (Figure 4d); also note that even if the B-γ phase is more stable at that temperature, its free energy difference with the B-α is negligible (2 meV per formula unit). This result apparently challenges experiments showing a spontaneous transformation of the B-γ into the Y phase at room temperature. However, such deterioration of the black perovskite has been observed only in samples exposed to air.^3^ Since CsSnI_3_ is synthesized as a powder, surface effects can be very sizable, and contamination of the sample with water and oxygen could alter the relative stability between the two phases. Moreover, it is known that the Y phase is easily oxidized and irreversibly transforms into the Cs_2_SnI_6_.^5,6^ Therefore, increasing the volume/surface ratio of the material (i.e., by growing larger crystals) would be a promising route to prevent the formation of the Y phase in the first place. The steep increase in the free energy difference between the two phases also shows how heating could efficiently remove contamination of the Y phase inside the solar cell.

In conclusion, we simulated the complete ambient pressure phase diagram of CsSnI_3_, showing an excellent agreement within 15% with the experimental transition temperatures for the black perovskite structures; this accuracy is comparable to the one of SSCHA+DFT (at the PBEsol level) in other materials where ionic fluctuations drive the phase transition rather than electronic processes^28−30^ and is achieved only through a complete treatment of anharmonicity. The lower free energy of the black perovskite structure compared to the yellow phase at room temperature is beneficial for the stability of the system, as preventing the formation of the yellow phase is an important step toward the stabilization of the perovskite structure in the air. Our approach is general and can be employed in any other metal halides, paving the way to a reliable high-throughput study of these materials with first-principles simulations.

## Methods

We studied the structure and the electronic properties of CsSnI_3_ within density-functional theory (DFT) in the PBEsol approximation,^18^ using the Quantum ESPRESSO distribution^31^ employing a plane-wave basis set, norm-conserving pseudopotentials from the Pseudo-Dojo library,^32^ and a cutoff of 70 Ry. The Brillouin zone for electrons is sampled with an 8 × 8 × 8 uniform mesh with respect to the primitive-reciprocal cell of the B-α structure; this sampling is appropriately rescaled in the other phases.

Anharmonicity and phonons are studied with the stochastic self-consistent harmonic approximation (SSCHA).^13,17^ SSCHA calculations are performed on a 2 × 2 × 2 supercell of the B-α phase containing 40 atoms; their convergence has been verified by comparing the results obtained in a 3 × 3 × 3 supercell with 135 atoms at one temperature (300 K), showing no significant differences. To converge the free energy and the thermodynamic properties (heat capacity and bulk modulus) with the supercell, we exploited the natural division of the SSCHA free energy into a long-range harmonic-like term and the short-range anharmonic correction.^17^ The harmonic-like free energy has been interpolated into an 8 × 8 × 8 supercell containing 2560 atoms, accounting also for long-range electrostatic interactions (LO–TO splitting). The same conditions were also applied to the Y phases.

To evaluate the thermodynamic properties and second derivatives of the free energy within the SSCHA, we introduce a new algorithm. The properties of interest for this work are related to entropy and pressure as
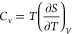
2
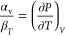
3
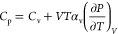
4where β_*T*_ is the isothermal compressibility (the inverse of the bulk modulus), and α_v_ is the volumetric expansion coefficient, shown in Figure 3. Thanks to correlated sampling,^19,33−35^ we can slightly vary the temperature at a fixed volume without the need for any new DFT calculation, obtaining the free energy and its derivatives (the entropy *S* and the pressure *P*) at temperatures surrounding the simulated one. We estimate the thermodynamic relations in eqs 2–4 by employing a finite-difference approach on the correlated sampling simulations (see the SI for more details).

The free energy Hessian needed to study the stability of the B-α phase reported in Figure 1 is computed as
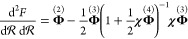
5where
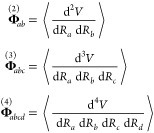
6and χ is the two-phonon free propagator as described in refs (13, 21, and 33). Usually, the four-phonon scattering tensor can be neglected in the inversion of eq 5(21,28,29,36−39) as it plays a negligible effect (bubble approximation). However, we found that its inclusion is necessary here to describe the subtle effects involved in the phase transition; otherwise, the B-α becomes unstable at all temperatures.

The spectral function reported in Figures 1 and 2 is defined as
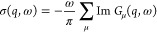
7where Im *G*_μ_(*q*, ω) is the imaginary part of the μth phonon dynamical Green function at *q*, evaluated with the time-dependent self-consistent harmonic approximation (TD-SCHA) nonperturbatively.^17^ The ω/π factor makes the integral proportional to the total number of modes.

In Figure 2 we report the full spectral function including the  term in the self-energy evaluated on the 40-atom supercell; this is made possible by the use of the Lanczos algorithm recently introduced.^17^ This cell is sufficient to get converged spectral function due to the short lifetime of phonons in this material. We choose the value of the smearing by checking the convergence with the supercell, possible only when neglecting the four-phonon scattering  in the self-energy with the interpolation introduced in ref (36).

## Supporting Information Available

Derivation of the expression of the heat capacity within the SSCHA theory and mathematical proof to eq 1 (PDF)
