# Supplementary material for: First-Principles Thermodynamics of CsSnI3
Source: Chem Mater. 2023 Feb 6;35(4):1702–9. doi: 10.1021/acs.chemmater.2c03475 (PMC9979598; doi:10.1021/acs.chemmater.2c03475)
Supplement: Supplementary file 1 — cm2c03475_si_001.pdf [file cm2c03475_si_001.pdf]

First-principles thermodynamics of $CsSnI_3$

Lorenzo Monacelli[1*] and Nicola Marzari[1]
[1]Theory and Simulation of Materials (THEOS), and National Centre for Computational Design and Discovery of Novel Materials (MARVEL), École Polytechnique Fédérale de Lausanne, 1015 Lausanne, Switzerland
[*] Email: lorenzo.monacelli@epfl.ch

Supporting Information

Heat capacity within the SSCHA theory

The heat capacity is the derivative of entropy with respect to temperature:

$$C_v = T\left(\frac{dS}{dT}\right)_v; \tag{1}$$

since the entropy is the derivative of the free energy

$$S = -\left(\frac{dF}{dT}\right)_v, \tag{2}$$

the heat capacity is proportional to the second derivative of the free energy with respect to temperature.

The entropy within the SSCHA is the entropy of the auxiliary harmonic Hamiltonian, i.e.

$$S(T) = k_b \sum_{\mu=1}^{3N} \left[-\ln(1 - e^{-\beta\omega(T)}) + \beta\omega(T)\frac{1}{e^{\beta\omega(T)} - 1}\right], \tag{3}$$

where $\omega_\mu(T)$ are the frequencies of the auxiliary harmonic Hamiltonian that solves the SSCHA at each temperature. The heat capacity is therefore

$$C_v = T\frac{dS}{dT} = T\frac{\partial S}{\partial T} + T\sum_{\mu=1}^{3N}\frac{\partial S}{\partial \omega_\mu}\frac{\partial \omega_\mu}{\partial T} = C_v^{(\text{harm})} + C_v^{(\text{anharm})}. \tag{4}$$

The first term is the standard specific heat of the quantum harmonic oscillator:

$$C_v^{(\text{harm})} = T\frac{\partial S}{\partial T} = k_b\sum_{\mu=1}^{3N}\frac{\beta^2\omega_\mu^2 e^{\beta\omega_\mu}}{(e^{\beta\omega} - 1)^2}, \tag{5}$$

whose high-temperature limit does not depend on the frequencies of the harmonic oscillator:

$$\lim_{T\to\infty} C_v^{(\text{harm})} = 3Nk_b. \tag{6}$$

This limit is reached exponentially fast above the Debye temperature of the highest frequency (in $CsSnI_3$ it is about $160\,\mathrm{cm}^{-1}$, equivalent to $230\,\mathrm{K}$); therefore, in all the simulation reported, this term is constant and equal to the high temperature limit. The intrinsic anharmonic contribution instead is:

$$C_v^{(\text{anharm})} = T\sum_{\mu=1}^{3N}\frac{\partial S}{\partial \omega_\mu}\frac{\partial \omega_\mu}{\partial T} = \sum_{\mu=1}^{3N}\frac{\beta\omega_\mu e^{\beta\omega_\mu}}{(e^{\beta\omega} - 1)^2}\frac{\partial \omega_\mu}{\partial T}; \tag{7}$$

$$C_v = C_v^{(\text{harm})} + C_v^{(\text{anharm})} = k_b\sum_{\mu=1}^{3N}\frac{\beta^2\omega_\mu^2 e^{\beta\omega_\mu}}{(e^{\beta\omega} - 1)^2}\left(1 + \frac{T}{\omega_\mu}\frac{\partial \omega_\mu}{\partial T}\right). \tag{8}$$

This expression gives the Eq. (1) reported in the main text in the limit $T \to \infty$.

Notably, the frequencies that appear in this equation are the SSCHA auxiliary frequencies, which are positive definite by construction, and never go to 0 (not even at a phase transition). Therefore, what causes the divergence of the specific heat at the phase transition is not the $1/\omega$ factor, but an inflection in the $\omega(T)$ curve. Thanks to this feature, the heat capacity remains well defined even below the transition, when the structure is metastable.
